# Identification and antibacterial evaluation of endophytic actinobacteria from *Luffa cylindrica*

**DOI:** 10.1038/s41598-022-23073-4

**Published:** 2022-10-29

**Authors:** Ramzy Ali Mahdi, Yadollah Bahrami, Elham Kakaei

**Affiliations:** 1 Department of Pharmaceutical Biotechnology, Faculty of Pharmacy, Kermanshah University of Medical Sciences, Kermanshah, Iran; 2 Medical Biology Research Center, Kermanshah University of Medical Sciences, Kermanshah, Iran; 3 Department of Medical Biotechnology, School of Medicine, College of Medicine and Public Health, Flinders University, Adelaide, SA 5042 Australia; 4 Department of Medical Biotechnology, Faculty of Medicine, Kermanshah University of Medical Sciences, Kermanshah, Iran

**Keywords:** Biological techniques, Biotechnology, Drug discovery, Microbiology, Molecular biology, Plant sciences, Medical research

## Abstract

The emergence of antibiotic-resistant bacteria has limited treatment options and led to the untreatable infections, thereby necessitating the discovery of new antibiotics to battel against bacteria. Natural products from endophytic actinobacteria (EA) serve as a reservoir for discovery of new antibiotics. Therefore, the current study focused on the isolation and antibacterial properties of EA isolated from *Luffa cylindrica*. Six strains were identified using morphological characterization, SEM analyses and 16S rRNA gene sequencing from the roots and leaves of the plant. They were taxonomically classified as *Streptomycetaceae* family. This is the first report on EA form *L. cylindrica*. The strains produced a chain of oval, cubed or cylindrical shaped spores with spiny or smooth surfaces. Three strains; KUMS-B3, KUMS-B4 and KUMS-B6 were reported as endophytes for the first time. Fifty percent of isolates were isolated from leaves samples using YECD medium. Our results showed that the sampling time and seasons may affect the bacterial diversity. All six strains had antibacterial activity against at least one of the tested bacteria *S. aureus, P. aeruginosa,* and *E. coli*. Among the strains, KUMS-B6 isolate, closely related to *S. praecox,* exhibited the highest antibacterial activity against both gram-positive and negative bacteria. KUMS-B6, KUMS-B5 and KUMS-B4 isolates strongly inhibited the growth of *P. aeruginosa.* Interestingly, the strains, isolated from leaves exhibited stronger antagonist activities compared to those isolated from the roots. The study revealed that the isolated strains from Luffa produce a plethora of bioactive substances that are potential source of new drug candidates for the treatment of infections.

## Introduction

Despite the amazing therapeutic effects of antibiotics against infectious diseases, the emergence of drug-resistant microorganisms has become a serious problem^1,2^. The baseless prescribing of antibiotics in addition to the inappropriate and arbitrary use of antibiotics has led to antibiotic-resistant bacteria, thus posing a significant challenge to prevention and treatment of infectious diseases. In the United States, over 2 million people are infected with antibiotic-resistant bacteria every year, causing 35,000 deaths annually. It is estimated that the global annual mortality rate due to antibiotic resistance will exceed 10 million by 2050^3^. Moreover, the emergence of pandrug resistance, a phenomenon characterized by bacteria becoming resistant to all classes of antibiotics, is also a growing concern^4^. Therefore, prevention and control of these microorganisms requires new strategies such as discovery and development of new drugs. Natural product screening is considered as the most promising line for novel antibiotic discovery, which is required to counteract the loss of effectiveness of the presently available antibiotics^5^. Plant-associated microorganisms have been widely used as sources of bioactive compounds for therapeutic purposes for many years^6,7^. Endophytic microorganisms reside inside plant tissues without causing obvious symptoms and damage and may include various groups such as fungi and bacteria, including actinobacteria^8^. Studies suggest a symbiotic interaction between host plants and endophytes in which the host provides shelter and nutrition, while endophytes act as chemical guards, promote plant growth, improve the host's tolerance for abiotic and biotic stresses, and increase disease resistance^9^. In addition, these endophytic actinobacteria may play crucial roles in maintenance and sustenance of specific natural habitats, but these endeavors call upon extensive scientific investigation to develop the necessary knowledge base^8,10^. Actinobacteria are generally free-living, Gram-positive filamentous bacteria with a high GC DNA content ranging from 50% to over 70%, found in aquatic and terrestrial habitats^9^. Over the last decades, actinobacteria, especially the genus *Streptomyces*, have been known to produce more than two-thirds of natural antibiotics (such as beta-lactams and tetracyclines). The discovery and use of antibacterials produced by actinomycetes is a new approach to deal with drug-resistant pathogens^11^. Fifty percent of 22,000 bioactive secondary metabolites of microbial origin is related to actinobacteria. Currently, about 160 antibiotics are used in the treatment of human infections and in agricultural industry^12^. Actinobacteria are known to be prolific producers of a wide range of bioactive natural products (NPs), and scientific evidence highlights their important protective role against diseases like colorectal cancer (CRC)^13^ and lung cancer^14^. Secondary metabolites of 17 Actinobacteria strains isolated from Antarctica had promising antitumor effects^15^. They also possess the potential to produce unique secondary metabolites, which can be exploited in pharmaceutical, agricultural and other industries^16^. Endophytic actinobacteria are critical resource of microbial biodiversity^17,18^. The taxonomic status of endophytic actinobacteria associated with medicinal plants has been investigated in several studies^17,19^. According to previous studies, almost all plants harbor endophytes. Endophytic bacteria can colonize various parts of the plant, including stems, roots, petioles, leaves, weed inflorescences, fruits, buds, seeds, along with dead and hollow hyaline cells. However, specificity and developmental stage of the host, geographical conditions and microbial diversity are variable factors that influence colonization of endophytes in different plants^8^. Search of particular ecological niches along with new methods of isolation of novel genera/species of actinobacteria may result in the identification of new gene clusters, and finally, new products^8^. Hence, the aim of present study was to explore the endophytic actinobacteria inhabiting special niches for discovering hitherto unexploited strains producing bioactive compounds. These strains can be used as an alternative source for the production of new antibiotics against pathogenic bacteria. For this purpose, *Luffa cylindrica* plant collected from different parts of Kerman, Iran. *L. cylindrica* is a medicinal plant utilized in traditional medicine for the treatment of cough, general body pain, asthma, tuberculosis, shingles, and boils. The fruits are anthelmentic, carminative, laxative, depurative, emollient, expectorant, tonic, and galactagogue and are useful in fever, syphilis, tumours, bronchitis, splenopathy and leprosy. This plant is mostly specific to the subtropical regions of Asia and Africa. Its fruits and leaves contain triterpenoid saponins^20,21^.

Many researchers have studied the antimicrobial properties of plant's endophytic actinobacteria^22,23^. There are plenty of reports on EA and their antibacterial activity against pathogenic bacteria. Their findings show a promisingly high probability of discovering a novel compound with different modes of action.

A few endophytic fungi including *Phialemoniopsis endophytica* sp have been reported from *L. cylindrica*^24^. However, to our knowledge, no study has been reported on the population of endophytic actinobacteria associated with *L. cylindrica* and this is the first report. Therefore, the aims of this study were to isolate and identify the endophytic actinobacteria from *L. cylindrica,* thus tapping into a still unexploited potential source of beneficial bacterial metabolites, and to determine their antimicrobial capability against the pathogenic bacteria; *Staphylococcus* aureus, *Pseudomonas aeruginosa*, *Escherichia coli*.

## Results

### Isolation of endophytic actinobacteria

**Figure 1:**
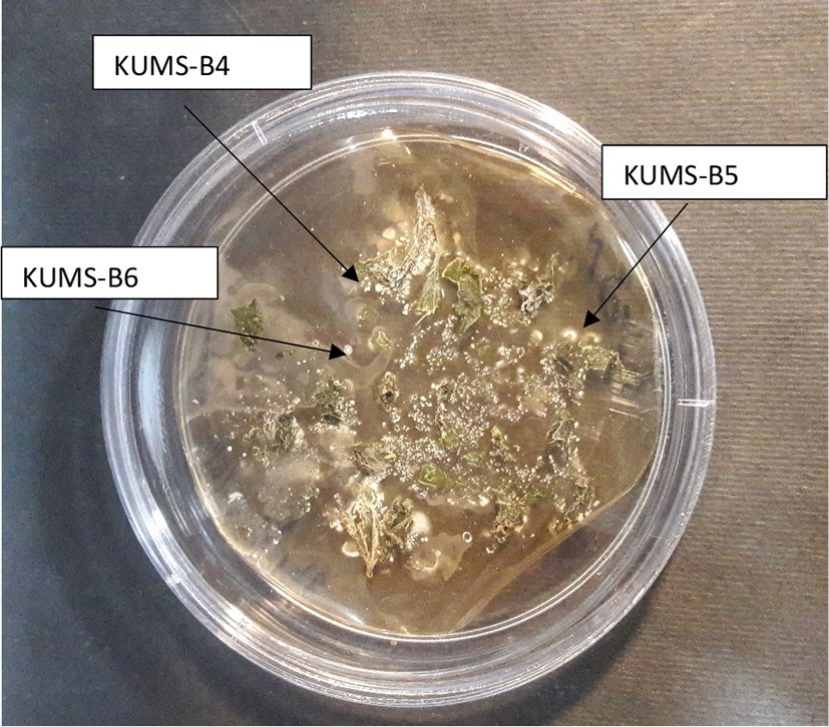
The emerging of *actinobacteria* from surface sterilized leaves following 6–8 weeks incubation on YECD medium. The arrows show colonies emerging from the surface-sterilized leaf tissue for KUMS-B4, KUMS-B5 and KUMS-B6 strains.

The surface sterilized samples were cultured on four different media and six strain having morphological characteristics of actinobacteria were isolated from different section of plant after 4–8 weeks of incubation onto TWYE medium at 28 °C. Figure 1 indicates actinobacterial hyphae rising from leaf fragments after 6 weeks of incubation onto YECD medium. The arrows show the growth of colonies from the leaves part of Luffa.

Six strains of endophytic actinobacteria, based on the morphological characteristics, were isolated from *L. cylindrica* using four different media. Three isolates (KUMS-B1, KUMS-B2, KUMS-B3) were isolated from the roots using TWYE culture medium and the other three isolates (KUMS-B4, KUMS-B5 and KUMS-B6) were isolated from the leaves using YECD culture medium. However, no actinobacteria growth was discerned on PDA or HPDA media. This is the first time to reported KUMS-B6 isolate as an endophyte based on morphological, microscopical features and 16S rDNA gene sequencing (Table 1). The isolates were cultivated onto ISP2 and their cultural and physiological features were determined using International Streptomyces Project (ISP2)^25,26^, used for characterization of strains.

**Table 1:** Identification of endophytic actinobacteria isolates from *Luffa cylindrica*.

Isolates	Morphological identification	Closest homologs (Similarity %)	Family	(EZbiocloud) (Similarity %)	GenBank (Similarity) (%)	Length	Accession no.
KUMS-B1	gram positive, Spherical creamy- pale brown colonies protruding surface, surrounded by a white border of spore, Fragmented mycelium, Soluble pigments	*S. koyangensis* (99.04%)	*Streptomycetaceae*	*S. albidoflavus* DSM 40455 (99.44)	*S. champavatii* NRRL B-5682 (99.45)	1455	ON626444
KUMS-B2	gram positive, Spherical gray- creamy colonies protruding surface, Non-fragmented mycelia,	*S. griseorubens* NBRC 12780 (99.86%)	*Streptomycetaceae*	*S. althioticus* (99.65)	*S. griseorubens* NBRC 12780 (99.86)	1477	ON626445
KUMS-B3	gram positive, Spherical creamy -brownish colonies protruding surface, Fragmented mycelia, White spores covered all colonies after two weeks,	*S. intermedius* NBRC 13049 (99.86%)	*Streptomycetaceae*	*S. intermedius* NBRC 13049 (99.86)	*S. intermedius* NBRC 13049 (99.86)	1475	ON626446
KUMS-B4	Small creamy-yellowish—colonies, Fragmented mycelia, gram positive	*S. microflavus* NBRC 13062 (100%)	*Streptomycetaceae*	*S.microflavus* NBRC 13062 (100)	*S. microflavus* NBRC 13062 (100)	1477	ON626447
KUMS-B5	gram positive, Spherical creamy white -light yellowish colonies, Fragmented mycelia,	*S. cavourensis* NBRC 13026 (99.86%)	*Streptomycetaceae*	*S. cavourensis* NBRC 13026 (99.86)	*S. griseobrunneus* NBRC 12775(100)	1480	ON626448
KUMS-B6	gram positive, Small white -brownish colonies, Fragmented mycelia,	*S. praecox* NBRC 13073 (99.80%)	*Streptomycetaceae*	*S. setonii* NRRL ISP-5322 (99.79)	*S. praecox* NBRC 13073 (99.80)	1477	ON626449

The table summarizes the accession no of isolates, and their homology, family and closely related stains.

**Figure 2:**
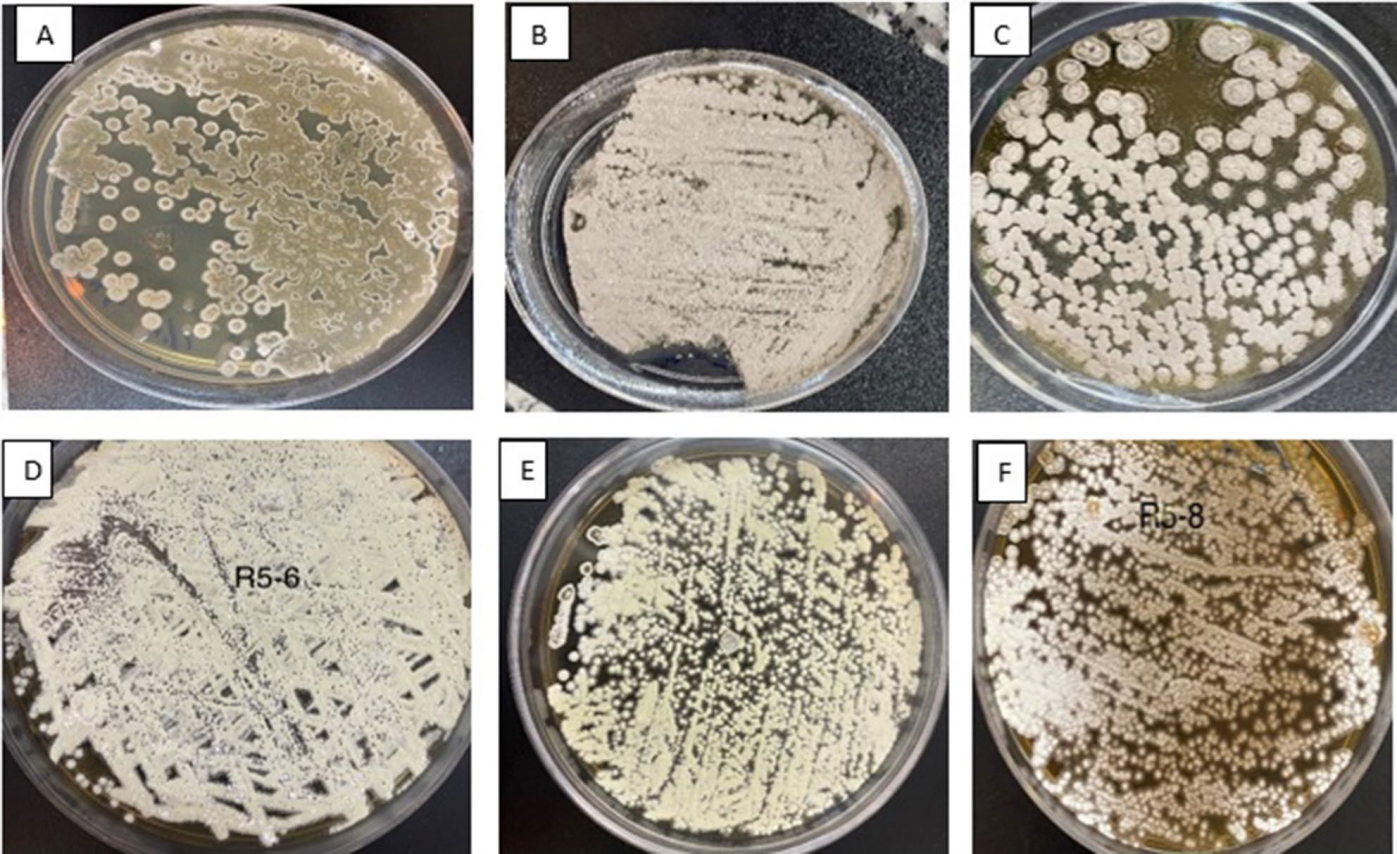
Morphological characteristics of six endophytic actinobacteria isolates from *Luffa cylindrica*, grown onto ISP2 after 2 weeks of incubation at 28 °C. KUMS-B1 isolate; cream–brown colony (**A**): KUMS-B2 isolate; grey–creamy watery colony (**B**) KUMS-B3 isolate; creamy-brownish colony (**C**): KUMS-B4 isolate; creamy–light yellowish colony (**D**): KUMS-C5 isolate; creamy–yellowish colony (**E**): KUMS-B6 isolate; small white–brownish colony (**F**).

Six endophytic actinomycetes were isolated from either root or leaf tissues, whereas no actinobacteria was isolated from fruits, stems and flowers. They were described based on colonial morphology onto ISP2, ability to generate aerial hyphae and substrate mycelia, and pigmentation as summarized in Table 1. The presence of aerial mycelium, spore mass colour, distinctive reverse colony colour, diffusible pigment, and sporophore and spore chain shape were studied based on the ISCC-NBS centroid colour system^27^. The actinobacteria colonies have white, creamy, pale yellow or brown to white brown and reddish colors after two weeks of incubation onto ISP-2, as demonstrated in Fig. 2. The morphological and structural characteristics of the isolates are listed in Table 1.

**Figure 3:**
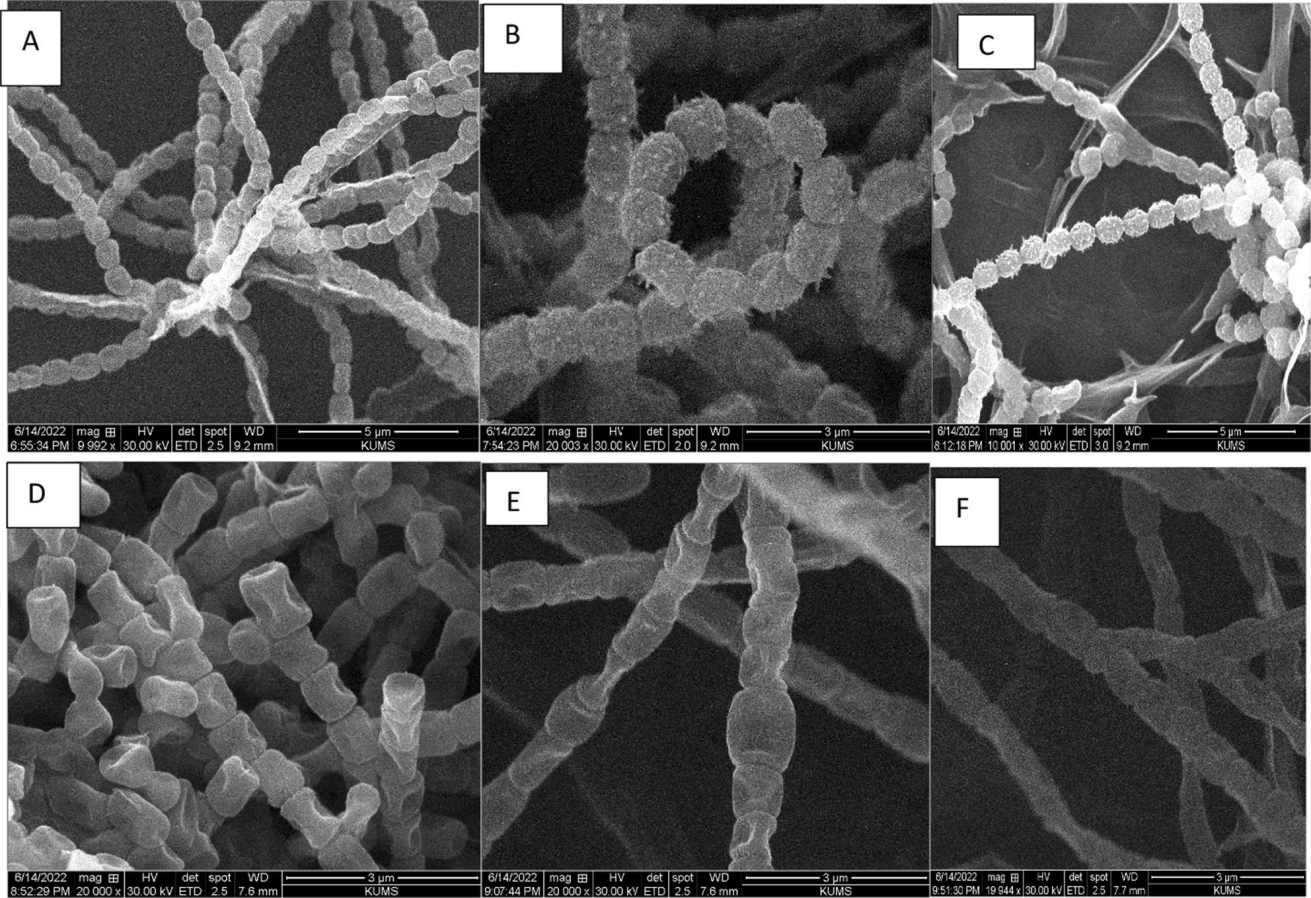
Scanning electron micrograph of mycelia and spores representing the spore-chain morphology and spore-surface ornamentation of strains and mycelia grown on ISP2 medium for 14 days at 28 °C at different magnification. *Streptomyces koyangensis* KUMS-B1 (oval and ovoid shape with smooth surfaces) (**A**); *S. griseorubens* KUMS-B2 isolate (flexuous spiral spore chains, oval and ovoid shape with warty, hairy or rough and coarse surfaces) (**B**); *S. intermedius* KUMS-B3 (straight spore chains, oval and ovoid shape with a warty, spiny or hairy surfaces) (**C**); *S. microflavus* KUMS-B4 (spores are cylindrical cube-shapes with indenting surfaces, and fragmented) (**D**); *S. cavourensis* KUMS-B5 (spores have cylindrical shapes with indenting surfaces) (**E**); *S. praecox* KUMS-B6 (The spores have cylindrical shapes with smooth surfaces) (**F**).

The morphology of spore shape, surface, and spore structure are important criteria for identifying actinobacteria. The morphology of spore chains and mycelia were also studied using SEM after two weeks growth on ISP2 medium at 28 °C. Scanning electron micrograph of mycelia and spores presenting the spore-chain morphology and spore-surface ornamentation of strains at different magnification are demonstrated in Fig. 3. The SEM results revealed that the aerial mycelia produced spore chains that were cuboid- spiral, cylindrical shaped, the branched and/or spiral or rod-shaped and/or long aerial mycelia and spore chains as demonstrated in Fig. 3.

Figure 3 shows spore-chain morphology and spore surface ornamentations of isolates after two weeks of incubation on ISP2 medium at 28 °C. They were clustered of single spores or spore chains. Spores of some actinobacteria had hairy surface and some had rugose or spiny surfaces. Generally, the spores were of oval and ovoid shape and had smooth surface (Fig. 3A), or coarse surfaces (Fig. 3B and C), but globes and rod-shaped spores, or cube shape such as KUMS-B4 (Fig. 3D) were also frequently found. The spores have cylindrical cube-shapes with indenting surfaces, which form in long straight branched chain ornamentations (Fig. 3D). Spores with spiny surface were observed with variable spine sizes (Fig. 3B and C). The spore chain of some strains such as KUMS-B2 and KUMS-B3 have hairy surface. Some are spiny and spiral chain such as KUMS-B2 (Fig. 3B). The spiral or flexuous spore chains were simple, open, loose coils of one to two turns, but some are hairy and in a long straight chain and branched ornamentations (Fig. 3C). Spore of some strains like KUMS-B1 have smooth surface. Strain KUMS-B2 showed a spore chain morphology of exhibiting spiral morphology.

### Molecular identification

#### PCR results

**Figure 4:**
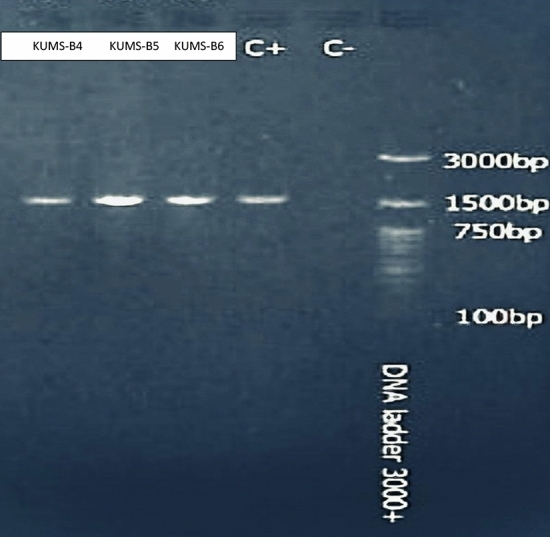
Gel electrophoresis of PCR products using 27F and 1492R primers. The 1.5 Kb amplified 16S rDNA fragments of isolated actinobacteria monitored on a 1.5% agarose gel. RCR product of 16S rRNA sequence of isolates on a 1.5% agarose gel from left to right: KUMS-B4, KUMS-B5, KUMS-B6, positive control, negative control and Ladder.

The genomic DNA was extracted, and 16S rDNA amplification performed to identify endophyte actinobacterial isolates. The results of the amplification of 16S rDNA fragments on 1.5% agarose gel electrophoresis are shown in Fig. 4. The original image of PCR gel electrophoresis is shown in Supplementary Fig. 1.

#### 16S rDNA gene sequencing

Endophytic actinobacteria strains were also identified by sequencing 16S rRNA gene segments. The sequences of isolates were aligned with those sequences of bacteria available in the datasets using the ClustalW and determined their similarity values. The presumptive relationships of these sequences were obtained from database comparison.

**Figure 5:**
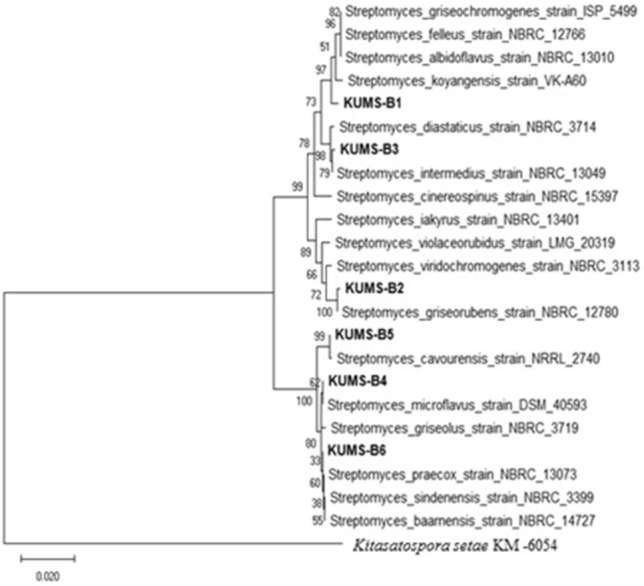
Phylogenetic tree of endophytic actinobacteria isolated from *Luffa cylindrica* constructed by the neighbour-joining method demonstrating their closely related type strains based on partial 16S rRNA gene sequences generated by MEGA X. The numbers at branch nodes show levels of bootstrap support (%) from 1000 replicates and scale bar refers to a phylogenetic distance of 0.02 nucleotide substitutions per site. *Kitasatospora setae* KM -6054 was used as outgroup.

The phylogenetic tree of the isolates was constructed based on the neighbour-joining algorithm with MEGA. The phylogenetic relationship of isolates based on 16S rRNA gene sequences is shown in Fig. 5. Based on 16S rRNA gene alignments and phylogenetic studies of isolates, they were categorized into one main cluster (Fig. 5). All identified isolates belonged to the *Streptomyces* genus, *Streptomycetaceae* family. They all belong to one distinct family within the Phylum *Actinobacteria*. According to our analyses the similarity of all isolates' 16 s rDNA sequences was more than 99% when compared to those of data presented in the dataset. It was possible to assign all the isolates to a species. *16S rRNA* gene sequencing revealed that endophytic actinobacteria represented six species; *S. koyangensis, S. griseorubens, S. intermedius, S. microflavus, S. cavourensis, S. praecox* (Fig. 5). The similarity, homology and accession numbers of the isolates with their closely related strains are summarized in Table 1. Among the examined isolates, KUMS-B4 had 100% similarity to *S. microflavus* NBRC 13062 in EzBioCloud and GenBank databases. The isolates had high percentage of similarity with their closely related strains, < 99% similarity to the existing database sequences of 16S rRNA genes. All the 16S rRNA gene sequences were submitted to GenBank and were assigned GenBank accession numbers ON626444, ON626445, ON626446, ON626447, ON626448, and ON626449.

#### Antibacterial effects of EA strains on pathogenic bacteria in solid-state culture

The set of test bacteria was used to determine the spectrum of antibiotic activity of strains. The antibacterial activity of ethyl acetate and methanolic extracts of the isolates, inoculated onto the solid culture was tested against Gram-positive and Gram-negative bacteria including *S. aureus* 25923, *P. aeruginosa* 27853, and *E. coli* 25922 using well diffusion assay. The results showed that the crude extract of the endophytic actinobacteria isolated from *L. cylindrica* were able to inhibit the growth of at least one of the test bacteria (Table 2). The test isolates displayed antibacterial activity against at least one of the target bacteria. In general, all strains had an inhibitory effect on at least one test bacteria. On the other hand, inhibitory activity was observed against all tested pathogens. Some strains showed antagonist activity against both gram-positive and gram-negative bacteria. The KUMS-B4, KUMS-B5 and KUMS-B6 strains showed a broad spectrum of antibiotic properties against all test bacteria, and demonstrated the largest inhibition zone against *P. aeruginosa* as compared to all isolated strains. Interestingly, all these strains were isolated from leaves. Ethyl acetate and methanolic extracts of KUMS-B4, KUMS-B5 and KUMS-B6 strains had inhibitory effects on the three tested pathogens. Ethyl acetate extracts of these three isolates showed the same inhibitory activity against *S. aureus.* Ethyl acetate extract of KUMS-B4 strain also showed a moderate effect on *E. coli* 25922, while KUMS-B5 and KUMS-B6 showed a stronger activity as compared to this strain. Methanolic extract of KUMS-B5 had a strong effect on *P. aeruginosa* 27853.

**Table 2:** Antibacterial effects of Endophytic actinobacteria isolated from *Luffa cylindrica* against tested bacteria.

Isolates	Methanolic crude extract			Ethyl acetate crude extract		
	*S. aureus* 25923	*E. coli* 25922	*P. aeruginosa* 27853	*S. aureus* 25923	*E. coli* 25922	*P. aeruginosa* 27853
KUMS-B1	−	+	−	-	−	−
KUMS-B2	+++	−	−	+	−	−
KUMS-B3	++	−	−	−	+	−
KUMS-B4	+	+	+	++	+	+++
KUMS-B5	++	++	+++	++	++	+++
KUMS-B6	+++	+	+++	++	++	+++

Imipenem and vancomycin were used as positive controls, while methanol and ethyl acetate were negative controls.

+++, strong activity; ++, medium activity; + weak activity and − no activity as compared to the positive controls.

The KUMS-B6 strain showed the strongest activity against target bacteria as compared to other isolates. Interestingly, the ethyl acetate and methanolic extracts of KUMS-B5 and KUMS-B6 strains had strong inhibitory effects against *P. aeruginosa* 27853. Ethyl acetate extract of KUMS-B4 was highly potent against *P. aeruginosa* 27853 and *S. aureus* 25923, while its methanolic extract had no a significant inhibitory activity against these bacteria. KUMS-B6 was active against all target pathogens making it as a promising source of broad-spectrum antibiotics that should be investigated further, while KUMS-B1 strain showed the weakest antimicrobial property so that only methanolic crude extract of this strain demonstrated a weak antimicrobial property against only one bacterium tested (*E. coli* 25922). Methanolic extract of KUMS-B1 strain was the only effective extract from this strain against *E. coli* 25922 and had no effect against other test pathogens. KUMS-B1 strain was shown the weakest antibacterial activity among 6 endophytic strains. From our findings, it seems that *S. aureus* was the most frequently inhibited target bacterium. No test bacteria showed an inhibitory zone with ethyl acetate extract of KUMS-B1 and KUMS-B2. This might indicate that there are no bioactive compounds in the extracts, having biocidal activities against test bacteria.

## Discussion

The antibiotic resistance is a crucial public health concern. Natural products have always been an important course of bioactive compounds namely antibiotics. They can be used to counteract the antibiotic resistance. Due to the increasing prevalence of multiple drug resistant pathogens (MDR) and widespread drug resistance (XDR) and problems related to the treatment and management of diseases caused by "ESKAPE" pathogens (*Enterococcus faecium*, *Staphylococcus aureus, Klebsiella pnumoniae, Acinetobacter baumanni, Pseudomonas aeruginosa*, and *Enterobacter* species), the development of new drugs is increasingly urgent^8,28,29^. Therefore, the use of diverse and newly endophytic actinobacteria isolated from particular habitats offers unique opportunities to extract new drugs and therapies. It is fascinating to explore and understand the biology and chemistry of endophytic actinobacteria and their host plants in order to develop new, non-destructive applications for human health, agriculture, and the environment^8,9^. Although the genus *Streptomyces* has been reported more frequently, the non-*Streptomyces* genus has also been reported as endophytes in different plants from harsh habitats^8^. Actinobacteria are one of the most important reservoirs of secondary metabolites with beneficial biological activities and also, one of the main manufacturers of common antibiotics^30^. The therapeutic properties of some of these secondary metabolites strongly suggest the abundance of potential new drug molecules in the vast chemical repertoire of endophytic actinobacteria that demonstrate plant protection and growth and, consequently, their importance in agriculture^8,19^. Sivalingam and colleagues (2019) reported the prominence of Actinobacteria, especially *Streptomyces* species derived from extreme sources, as a remarkable sources of new biosynthetic gene clusters with potential for anticancer drug development^31^. Actinomycetes are considered as a rich source of natural products, which have numerous bioactive functions, including phytotoxic, antimicrobial, insecticidal, and mainly antiproliferative and antitumor activities^32–37^. This is, to our knowledge, the first report on the diversity and antimicrobial activity of EA isolated from *L. cylindrica*. As with other reports, the present study also found that high-G + C Gram-positive endophytic bacteria were dominant in the luffa with streptomyces being the most abundant group. These observations were similar to the study conducted by Govindasamy et al. They isolated and identified endophyte actinobacteria from cactus (*Opuntia ficus-indic*a) for the first time^38^.

The present study for the first time addressed that *L. cylindrica*'s root and leaves are rich in endophytic actinobacteria, which six endophytic actinobacteria were isolated from these samples using cultivar-dependent method. Many studies performed on endophytic actinobacteria populations using culture-independent and culture-dependent methods. For example, Sessitsch et al. identified several species of *Streptomyces* in potatoes using PCR for16S rRNA gene followed by denaturing gradient gel electrophoresis (DGGE)^39^. There are many studies on endophytic actinobacteria isolation by which several genera such as *Streptomyces, Microbispora, Micromonospora* and *Nocardioides* have been isolated and reported from different plants^40^. In the present study based on the morphological characteristics and also partial 16S rRNA gene sequencing, six endophytic actinobacteria strains were isolated from *L. cylindrica*. Four different selection media were consumed to isolate actinobacteria. Three isolates were isolated from the roots (KUMS-B1, KUMS-B2, KUMS-B3) sampling from November 2020 and the other three isolates were isolated from the leaves (KUMS-B4, KUMS-B5 and KUMS-B6) sampling from September. The results showed that the sampling time and seasons may affect the results and bacterial diversity. The result also revealed that the leaves and roots are good habitats for EA. Fifty percentage of strains were isolated from leaves and the other 50% isolated from roots of the plant. We were unable to identify any EA from the fruits, stems and flowers. This might result from the intrinsic feature of plant species which are herbaceous, not woody plants. They are more vulnerable to the surface sterilization reagents and thus suggested that these substances might have penetrated to the samples and perished EA. The soft internal tissue and having a fibrous spongy skeleton network inside the fruits allow the penetration of sterilization reagents. In addition, decreasing the incubation time of surface sterilization reagents might lead to the isolation of EA from these tissues. Three isolates were isolated from TWYE culture medium (KUMS-B1, KUMS-B2, KUMS-B3) and three isolates (KUMS-B4, KUMS-B5 and KUMS-B6) from YECD culture medium. No growth of actinobacteria was detected on other selected media used. The results unravel the importance of using a specific culture medium as well as the plant sampling site to isolate different species of endophytic actinobacteria. According to morphological analysis, all isolates belonged to *Streptomycetaceae* family. Fragmented mycelium was observed for all isolates. The appearance of the KUMS-B1 colonies on ISP-2 medium was spherical, wrinkled, and protruding with a cream-colored bald surface, which was surrounded by a white layer of spores. The KUMS-B2 colonies into ISP-2 medium was spherical and protruding with cinereous, dove-grey color and watery surface, but smaller than KUMS-B1 colonies. The KUMS-B3 colonies was also spherical and protruding with the creamy-white and fuzzy surface. White spores covered all colonies after two weeks. The KUMS-B4 colonies into ISP-2 medium was small spherical with cream-yellowish color. In this strain, spore production was faster. The KUMS-B5 colonies was full of colorful creamy- light yellowish colour, while the KUMS-B6 produced small colonies with white-brownish color into ISP-2 medium. Spore production was done in this strain at a lower speed than other strains. Similar to our results, in a study conducted by Chromkaew's Shutsrirung et al. the most frequent isolates recovered were members of *Streptomyces*^41^.

The spore-chain morphology and spore surface ornamentations are crucial for identification of actinobacteria. Actinomycete spore chain length, shape, position, color are the important basis for classification^5^. The growth rate and sporulation rate of each isolate varied on ISP2 medium. The strains had clear and unique pattern of spores’ arrangement. The spore surface of the isolates varied from smooth and fluffy to rough and flattened and indented. Some isolates from root had oval and ovoid -shaped spores with spiny and hairy surfaces, while isolated strains from leaves had a chain of cylindrical or cubed shape spores with smooth and/or indented surfaces. Our results are in agreement with findings of Riquelme^5^ and Ali et al^42^. We used inclined glass coverslip technique and acquired the good result, as describe by Kurtboke^43^ as the best technique. The concept of smooth, warty, spiny, and hairy spore surfaces has been used for characterization of actinobacteria.

*16S rRNA* gene sequencing revealed that endophytic actinobacteria represented six species; *S. koyangensis, S. griseorubens, S. intermedius, S. microflavus, S. cavourensis, S. praecox*. and exhibited 99% similarity with their closely related actinobacterial strains. The present study indicated all the six isolates belong to same phylum *Actinomycetota* and one genus, *Streptomyces,* which is consistent with other studies, reported mainly from *Actinomycetota* phylum^22,41^.

Three strains, closely related to *S. microflavus*, *S. intermedius* and *S.praecox* were reported as endophytes for the first time and they were isolated from leaf and root samples using YECD and TWYE culture media.

Numerous factors can affect the diversity of endophytes, such as plant age, plant sampling site, soil type, seasonal collection, geographical location, and other environmental conditions^44,45^. The similarity of strains with their closely related stains, deposited in GenBank and other databases is more that 99%. The closely related strain to KUMS-B4 strain in the GenBank and EZbiocloud databases was found to be *S. microflavus* strain NBRC 13062, which had 100% similarity in sequence. This is first to report *S. microflavuas* as an endophyte*.* It was originally isolated from soil and recently from marine environment^46^. According to the phylogenetic tree, KUMS-B5 was closely related to *S. cavourensis* NBRC 13026. *S. cavourensis* YBQ59, and *S. cavourensis* strain 1AS2a reported as endophytes^47,48^. Based on the phylogenetic tree, KUMS-B6 strain was placed next to *S. praecox* strain NBRC 13073, which had 99.80% similarity in sequence. Besides, the KUMS-B1 strain was placed next to *S. koyangensis* VK-A60, which had 99.04% sequence similarity. The *S. koyangensis* strain B025 reported as endophytes^49^. The 16S rRNA gene sequence of strain KUMS-B3 showed the highest sequence similarity with *S. intermedius* NBRC 13049, reported from soil. Nevertheless, further investigations of strains should be carried out to determine their correct phylogenetic placement.

The present study demonstrated the antibacterial effect and inhibitory potential of endophytic bacteria isolated from *L. cylindrica* against test bacteria. The results revealed that the crude extracts of the isolates were able to inhibit the growth of at least one of the test bacteria due to the presence of natural bioactive compounds with growth-inhibitory properties^8,50^. This finding is consistent with other studies, which reported the endophytic actinobacteria as a potential source of secondary metabolites containing bioactive compounds^50,51^. Some strains such as KUMS-B6 isolate had antagonist activity against both gram-positive and gram-negative bacteria. The KUMS-B6, isolated for the first time as endophyte, exhibited the highest antibacterial activity against all test bacteria, highlighting this strain as new potential source for a wide range of antibacterial agents. Zarandi, et.al. studied the antagonist activity of *S. praecox* strain R7 to control *Rhizoctonia solani* in tomatoes. They stated that *S. praecox* enhances the expression of lipoxygenase and phenylalanine ammonia lyase by triggering jasmonic acid and phenyl propanoid signaling pathways in plants; therefore, resulting an induced defense status in tomatoes against *R. solani*^52^. Interestingly, KUMS-B6, KUMS-B5 and KUMS-B4 isolates also strongly inhibited the growth of *P. aeruginosa*. A few compounds; chromomycin A9, chromomycin Ap, chromomycin A2, and chromomycin A3 were reported from the ethyl acetate extract of *S. microflavus* which showed potent antibacterial activities against Gram-positive bacteria^46^ which are consistent with our findings. Four metabolites; (2E, 4E) -5- (3-hydroxyphenyl) -penta-2,4-dienamide, Ergosterol, Ergosterol peroxide and Halolithuralin B, were documented from *Streptomyces* sp. YIM65484 to have antibacterial activity^53^. In addition, eight compounds; 1-monolinolein, bafilomycin D, nonactic acid, daidzein, 3'-hydroxydaidzein, 5,11-epoxy-10-cadinanol, prelactone B, and daucosterol were identified from *S. cavourensis*YBQ59 exhibited inhibitory activity against Gram positive bacteria^47^ which is in agreement with our findings. Mondal and Rai et al., also reported antibacterial activity of endophytic *S. cavourensis* MH16 isolated from *M. hortensis* against Gram-positive and negative bacteria^54^. Among the isolates, KUMS-B1 strain showed the weakest antimicrobial properties. This strain had no antibacterial activity on test bacteria or showed a weak antimicrobial property against *E. coli* 25922. The antifungal activity of 4-phenyl-3-butenoic acid from *S. koyangensis* VK-A60, isolated from soil^55^ was reported by Lee et al.^56^. The KUMS-B2 strain had 99.86% similarity to *S. griseorubens* strain NBRC 12780. *S. griseorubens* MPT42 has already been isolated as an endophyte from *Litsea cubeba*^57^. This strain reported to expresses all three biosynthetic genes associated with the antibiotics PKS-I, PKS-II and NRPS and has shown significant antimicrobial activity^57^. The antimicrobial properties of this strain have been demonstrated in many studies^58,59^. Methanolic extract of KUMS-B2 strain had a great inhibitory effect against *S. aureus* 25923. The antibacterial compounds generated by this strain have probably polar structures and the Gram-positive bacteria generally were more susceptible to extracts from this strain as compared to gram-negative bacteria. The influential difference may be due to the structure of their cell walls^60^. Similarly, Ameen, et al.^61^ reported the antibacterial activity of thirty endophytic actinobacterial strains against antibiotic-resistant bacteria. Ethyl acetate extracts of actinobacteria inhibited the growth of Gram positive and negative bacteria^62,63^*.* The methanolic extract of KUMS-B3 had a moderate inhibitory activity against *S. aureus*. This is the first antibacterial report on closely related strain to *S. intermedius.* The present study for the first time indicated that *L. cylindrica* is rich in endophytic bacteria where six endophytic bacteria were isolated from roots and leaves samples and all the isolates belonged to same phylum *Actinomycetota* and represented one genera *Streptomyces*. In general, the strains, isolated from leaves, exhibited stronger antagonist activities against test bacteria as compared with those, isolated from roots. Therefore, they are more promising sources for discovery of novel antibacterial compounds and industrial applications. In this study, *S. praecox* was isolated as endophyte for the first time, exhibited the highest antibacterial activity against all three tested bacterial pathogens.

## Methods

### Samples collection and authentication

Symptomless and mature *L. cylindrica* plant collected from different parts of Kerman, Iran (southeast of Iran, 28.6751° N, 57.7/372° E). The samples collected in September and November 2021 to cover seasonal variation. All the procedure related to plants were complied with relevant institutional, national, and international guidelines and legislations. The collection of specimens was done randomly under the Research Center of Agriculture and Natural Resources of Kermanshah (RANK) permit, provided by the governments. They were then identified and authenticated by an expert, Dr. Nastaran Jalilian at the Kermanshah Agricultural and Natural Resources Research and Education Centre, Kermanshah, Iran according to the Flora of Iran and Flora Iranica. The voucher specimen (10395) was deposited in the Herbarium of RANK. The plants were dissected to stems, leaves, roots, and, fruits, and surface-sterilized and cultured within 48 h of sampling.

### Surface sterilization

The surface sterilization of plant samples (roots, stems, leaves and fruits) was performed according to procedure described by Kaewkla and Franco^64^ and Ali et al^42^. Breifly, the plant tissues were washed with running tap water to remove all contamination and any physical debris. The samples were cut into pieces using a sterile saw/knife and tweezers and dried in natural air after washing three times with distilled water. The segments were cut to create small pieces of two to five cm length and their surfaces were sterilized with various surface disinfectants to prevent the growth of bacteria and non-endophytic fungi. Each sample was immersed in sterile 0.1% Tween 20 for five minutes. The samples were then treated with 70% ethanol for five minutes, and then the samples were rinsed with freshly prepared 6% sodium hypochlorite (NaOCl) solution. Samples were washed several times with DDW to remove chemical residues and immersed in sterile 10% (w/v) sodium bicarbonate (NaHCO_3_) for 10 min to delay the growth of endophytic fungi^65^ and were washed three times with sterile distilled water and dried in a laminar flow. In order to ensure the efficacy of the surface sterilization, 100 µL of the last double distilled washed water was cultivated onto ISP-2 media and incubated at 28 °C for 1 week. The absence of microbial growth on the culture media after plating the last washing water demonstrated the effectiveness of surface sterilization^66^.

### Isolation of endophytic actinobacteria

To isolate the endophytic actinobacteria, HPDA, PDA (Merck, Germany)^67^, TWYE (Merck, Germany)^68^, and YECD (Merck, Germany)^69^ media supplemented with nalidixic acid (25 µg/mL) and benomyl (10 µg/mL) to inhibit the growth of gram negative bacteria and fungi were used^70,71^. Then the surface sterilized samples were aseptically cut to 1–2 cm, placed directly on the culture medium (in triplicate), and incubated at 28 °C for 16 weeks. The plates checked periodically and the growth of endophytic actinobacteria colonies from the tissues monitored daily, up to 16 weeks. Different colonies with putative actinobacteria characteristics were introduced and sub-cultured onto international *Streptomyces* Project-2 medium (ISP2)^72^. Pure cultures obtained after two to three consecutive subcultures and transferred to fresh isolation media, considering the possibility that each colony resembles a distinct strain^73^. Finally, the stock culture was prepared using Tryptic Soybean Broth (TSB) (Merck, Germany) containing 30% glycerol from the strains cultured on the ISP2 medium and stored at − 20 °C for further analysis.

### Identification of endophytic actinobacteria

The isolates were identified according to their morphological characteristics^19^ such as, colony features, the presence of vegetative and reproductive mycelium, fragmentation of the mycelium, spore production, and pigments, following the general guidelines of the International Streptomyces Project. In addition, Gram staining and Scanning Electron Microscope (SEM) analysis (FEI, Quanta 450, America) were used to confirm the isolates^74,75^.

### Scanning electron microscopic (SEM) examination

The morphology of isolates was also studied using SEM (FEI, Quanta 450, America). The isolates were cultured onto ISP2, the aerial mycelium and spores were obtained on a cover slip /slide. Cover slides were cut into one cm pieces and sterilized. The Sterile coverslips were inserted into solidified ISP2 at an angle of 45°. The inoculum was spread along with the glass-agar medium interface and incubated at 28 °C for 14 days. During incubation, the organisms grew over the surface of the glass pieces. The cover slip containing organisms was then coated with a film of gold about 150–200 A° thickness and observed under SEM at an accelerating voltage of 25.000–30.000 V for spore surface ornamentation^74^. The spore chain morphology and ornamentation of the potential strains were analyzed.

### DNA extraction

Molecular identification of isolates was carried out using *16S rRNA* gene sequencing to identify selected endophytic actinobacteria species. The genomic DNA was extracted from the endophytic actinobacteria according to Coombs et al. protocol^69^ using TSB broth medium. For this purpose, first, under sterile conditions the bacterial colony was inoculated into Erlen Meyers containing 25 mL TSB and incubated for 7 days in a shaker incubator at 28 °C (HYSC, Korea) at 160 rpm. After about 7 days, the purity of cultures was monitored using wet mount and Gram staining methods by a light microscope (Olympus). The supernatant was then removed with a Hettich 320R, Germany (4,000 rpm, 10 min, 4 °C) to separate the mycelia, and 50–100 mg of bacterial pellets were used to DNA extraction and poured into 2 mL microtubes and added 500 µL TE (Tris 10 mm, EDTA 1 mm), vortexed and then centrifuged, (3 min, 5000 rpm). Then, 10 µL of lysosome (10 mg/mL) was added to each microtube and incubated for 60 min at 37 °C. Ten µL of proteinase K (10 mg/mL) and 32.5 µL of SDS 10% were added to the microtubes and incubated for 60 min at 55 °C. After that 100 µL NaCl 5 M and 65 µL CTAB/NaCl were added to the microtubes and incubated ten minutes at 55 °C. Then, 600 µL chloroform-alcohol alcohol (24:1) was added to the microtubes and after a short vertex, incubated 30 min at room temperature, and then Centrifuged, at 12,000 rpm for 15 min. The supernatant was transferred to the new microtubes and 500 µL chloroform was added, and incubated at room temperature for 15 min and then centrifuged for 15 min at 12,000 rpm. The supernatant was transferred to the new microtubes and added 10 µL RNAase (10 mg/mL), incubated for 60 min at 37 °C and then 55 °C for 5 min to inactivate the RNase enzyme. After that, washed with chloroform. The supernatant was treated with 1 × volume of 3 M sodium acetate (50 µL) and 3× volume of 100 percent cold ethanol (1 mL) and kept in a freezer at – 20 ℃ overnight. Following centrifugation for 15 min at 15,000 RPM, the supernatant was transferred to the new microtube and 500 µL ethanol 70% was added and centrifuged 15 min at 8000 rpm. The supernatant was discarded and the pellet was dried using a heat block. Finally, 50 µL of deionized distilled water was added to the sediment and the microtube was preserved in – 20 ℃. Agarose gel electrophoresis and NanoDrop 2000c spectrophotometer (Thermo Scientific, USA) were used to evaluate the quality of the extracted DNA.

### Molecular identification of isolates

#### PCR

The 16S rRNA genes were amplified by PCR as described by Coombs and Franco using Mastercycler® nexus, Eppendorf and primers namely 27F (AGAGGTTGATCMTGGCTCAG) and 1492R (TACGGTACTTTTTACTACTTT)^40^. Briefly, the amplifications were carried out in a total volume of 25 µL reaction mixture, including 12.5 µ of master mix (Sinaclon Cat.No.MM2062), 0.3 µL of each primer (10 pmol/μL), 1 µL of DNA template (50–100 ng), and 10.9 µL of D.W. Amplification was performed in a thermocycler (Mastercycler® nexus, Eppendorf). The thermocycle program was 94 °C for 4 min, followed by 30 cycles of 94 °C for 1 min, 50 °C for 2 min and 15 s and 72 °C for 2 min, and finally an extension step at 72 °C for 5 min. The PCR product was then confirmed by gel electrophoresis and UV-translummant device (Gel Doc device (Vilber, France). PCR products were sequenced by a commercial company, Macrogen, South Korea.

#### Molecular phylogenetic analysis

The Partial 16S rRNA gene sequences were compared with the sequences retrieved from EzBioCloud and GenBank, NCBI databases using BLASTn tool (Basic Local Alignment Search Tool) (http://www.ncbi.nlm.nih.gov/BLAST/Blast.cgi)^76^. Multiple sequence alignment was performed using the CLUSTAL W 1.83 program, and a phylogenetic tree was reconstructed via the bootstrap test of neighbor-joining method using MEGA X (Molecular Evolutionary Genetics Analysis) software^19^. The Kimura two-parameter model was used to calculate pairwise distances for the neighbour-joining procedure (Kimura 1980). Strength and reliability of internal branches (topologies of the neighbor- joining) of the resultant tree was assessed after a 1000 bootstrap -replicate analysis^77^. *Kitasatospora setae* KM -6054 was used as an outgroup strain for tree constructions^78^. The partial 16S rRNA gene sequences, determined in the present study, were deposited in GenBank under accession numbers listed in Table 1.

#### Metabolites extraction

To test the antibacterial activity of endophytic isolated strains, the secondary metabolites from the isolates were extracted using ethyl acetate and methanol, respectively. Briefly, Isolates were grown for 7 days on plates (10 cm in diameter) containing TSA medium at 28 ± 2 °C. After that, they were cut into small pieces with a razor blade and then poured into a 100 mL pre-sterilized Erlenmeyer. Then 50 mL of ethyl acetate was added to the Erlenmeyer flakes and place on a shaker (180 rpm) for 24 h at room temperature. The ethyl acetate was then filtered and transferred to the Falcon 50 mL. Then we added 50 mL of methanol to the pellet and allowed the device to operate for another 24 h under the same conditions. After that, the methanolic extracts were filtered. A rotary evaporator (Rotary Evaporator RE100, Heidolph, Germany) (70 RPM, 38 °C) was used to concentrate the extracts. The extracts were stored at − 20 °C^79^ until used.

#### Antimicrobial activity of isolated strains

The antibacterial activity of the isolates (KUMS-B1, KUMS-B2, KUMS-B3, KUMS-B4, KUMS-B5, and KUMS-B6) was screened against three pathogenic bacteria (*Escherichia coli* ATCC 25922, *Staphylococcus aureus* ATCC 25923 and *Pseudomonas aeruginosa* ATCC 27853), purchased from microbial Bank of Pasteur Institute of Iran, using agar well diffusion method. Initially, the test bacteria were inoculated in TSB medium at 37 °C for 24 h. The growth was adjusted to half the McFarland's dilution (OD = 0.2) using Spekol 1500 spectrophotometer at 600 nm (OD600 nm). Then, the antibiotic agar medium (AAM-MERCK) was seeded with the tested culture (1% V/V) and dispensed into petri dish plates. After that, 10 wells with a diameter of 6 mm were prepared using a sterile cork borer with specified intervals in each plate. Each well was than loaded with 50 µL of extracts, allowing the wells to dry completely. Finally, the plates incubated at 37 °C for 24 h. Vancomycin (20 µg/mL) and Imipenem (15 µg/mL *P. aeruginosa* ATCC 27853 and 1.5 µg/mL *E. coli* ATCC 25,922) were used as positive controls for Gram-positive and Gram-negative bacteria, respectively. Wells containing methanol or ethyl acetate (50 mL) were used as negative controls. Each test was repeated thrice and the zone of growth inhibition around each well was measured and its average was recorded in mm.

## Conclusion and future prospective and directions

This is the first study screening the EA from *Luffa cylindrica*. The study revealed that *L. cylindrica* harbors untapped diversity of endophytic actinobacteria with valuable antimicrobial potential. All isolates belong to the family *Streptomycetaceae* in which three of those reported as endophytes for the first time. We analyzed limited numbers of strains based on 16S rRNA gene sequences which need further analysis regarding their PKI and NRPs. To understand the chemical structure of antimicrobial compounds, further analytical purification of extracts is required to decipher their mechanism of action.

Interestingly, some isolates were able to inhibit the growth of both gram-positive and gram-negative pathogens and reveal the possible presence of broad-spectrum of antibacterial compounds. These bioactive substances can be considered as a potential source for the synthesis of new antibacterial drugs for the treatment of infectious diseases, but more studies are required to isolate and identify these bioactive substances.

Our finding along with literature highlights that actinobacteria are able to produce a diverse range of secondary metabolites, which should be explored further for new antimicrobial drug candidates. These data may have significant implications for the antibiotic resistance, health and pharmaceutical applications and are particularly relevant to the development of antibiotic agents.

## Supplementary Information

Supplementary Figure 1.

## Data Availability

The following accession numbers have been assigned to the nucleotide sequences generated during the current study, deposited in the GenBank databases: ON626444, ON626445, ON626446, ON626447, ON626448, and ON626449.
