# Supplementary material for: Identification and antibacterial evaluation of endophytic actinobacteria from Luffa cylindrica
Source: Sci Rep. 2022 Oct 29;12:18236. doi: 10.1038/s41598-022-23073-4 (PMC9617871; doi:10.1038/s41598-022-23073-4)
Supplement: Supplementary file 1 — Supplementary Figure 1. [file 41598_2022_23073_MOESM1_ESM.pdf]

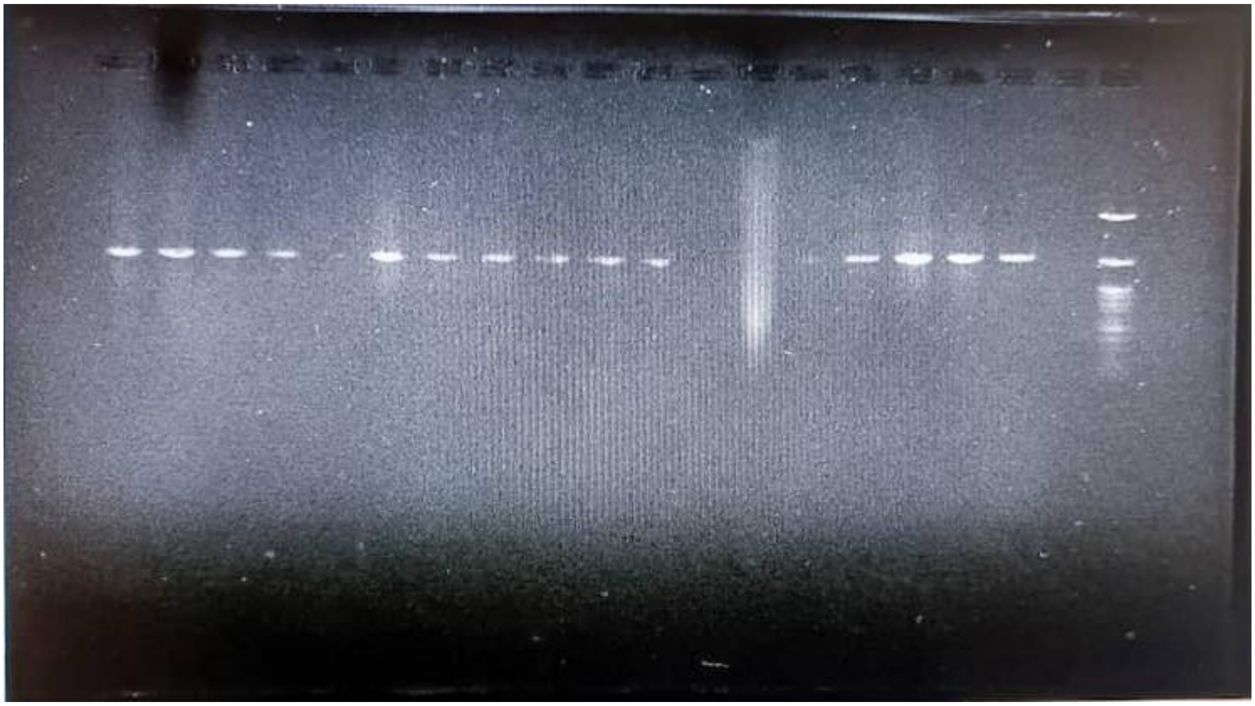

Supplementary figure 1; The whole gel of Fig. 4. Gel electrophoresis of PCR products using 27F and 1492R primers. The 1.5 Kb amplified 16S rDNA fragments of isolated actinobacteria monitored on 1.5% agarose gel. The whole gel of RCR product of 16S rRNA sequence of isolates on 1.5% agarose gel. From right to left: Ladder, negative control, positive control, KUMS-B6, KUMS-B5 and KUMS-B4.
